# MiR-30c-5p ameliorates hepatic steatosis in leptin receptor-deficient (db/db) mice via down-regulating FASN

**DOI:** 10.18632/oncotarget.14561

**Published:** 2017-01-09

**Authors:** Jiahui Fan, Huaping Li, Xiang Nie, Zhongwei Yin, Yanru Zhao, Chen Chen, Dao Wen Wang

**Affiliations:** ^1^ Division of Cardiology, Department of Internal Medicine, Tongji Hospital, Tongji Medical College, Huazhong University of Science and Technology, Wuhan 430030, China

**Keywords:** miR-30c-5p, NAFLD, hepatic steatosis, FASN, db/db

## Abstract

Approximately 15–40% of the general adult population suffers from non-alcoholic fatty liver disease (NAFLD) worldwide. However, no drug is currently licensed for its treatment. In this study, we observed a significant reduction of miR-30c-5p in the liver of leptin receptor-deficient (db/db) mice. Remarkably, recombinant adeno-associated virus (rAAV)-mediated delivery of miR-30c-5p was sufficient to attenuate triglyceride accumulation and hepatic steatosis in db/db mice. Through computational prediction, KEGG analysis and Ago2 co-immunoprecipitation, we identified that miR-30c-5p directly targeted fatty acid synthase, a key enzyme in fatty acid biosynthesis. Moreover, down-regulation of FASN by siRNA attenuated some key features of NAFLD, including decreased triglyceride accumulate and lipid deposition. Our findings reveal a new role of miR-30c-5p in counterbalancing fatty acid biosynthesis, which is sufficient to attenuate triglyceride accumulation and hepatic steatosis in db/db mice.

## INTRODUCTION

With the obesity pandemic, non-alcoholic fatty liver disease (NAFLD) has become the most common chronic liver disease affecting 15–40% of the general adult population and an important cause of cirrhosis and hepatic carcinoma worldwide [1–3]. NAFLD is composed of a series of pathological changes in liver: 1) The initial stage is hepatic steatosis, characterized by the excess deposition of triglyceride and/or cholesterol in liver; 2) Abnormal hepatic lipid accumulation results from augmented de novo lipogenesis, and/or decreased β-oxidation; 3) If uncontrolled, hepatic steatosis can progress to cirrhosis, end-stage liver failure and hepatocellular carcinoma [4, 5]. Currently, no drug is licensed for the treatment of NAFLD [3, 6], and thus it is important to understand the mechanisms underlying NAFLD and develop new intervention strategies.

MicroRNAs (miRNAs) are a class of endogenous, small, non-coding RNAs, which emerge as powerful regulators in many essential biological processes [7]. MiRNAs suppress gene expression through two major mechanisms: translational repression or mRNA cleavage/degradation, depending on the ability to bind their mRNA targets [8]. Due to their extraordinary variability of expression patterns and functions across tissues and physiological/pathophysiological states, miRNAs can be powerful diagnostic and therapeutic tools in various disorders, such as cancer, neurological diseases, cardiovascular diseases and metabolism disorders including NAFLD [9–12]. Specifically, up-regulation of miR-125b by estrogen protects against NAFLD in female mice [13]. MiR-21 decreases the levels of triglyceride and cholesterol by targeting HMGCR [14]. Thus, these studies have raised the enthusiasm to explore the roles of miRNAs in NAFLD.

Our published data discovered dysregulated circulating miR-30 family in patients with high risk factors of coronary artery disease, such as hyperlipidemia [12], which indicated that miR-30 family might be metabolism related miRNAs. Though the same seed sequence is shared by all the members of the miR-30 family, miR-30c-5p possess the highest abundance in liver, compared with other miR-30s (our unpublished data). While previous work demonstrated that miR-30c reduced atherosclerosis in *Apoe*^−/−^ mice [15], the role of miR-30c-5p in NAFLD remained elusive.

In the present study, we found that miR-30c-5p was significantly decreased in liver of leptin receptor deficient (db/db) mice, a classic animal model for liver steatosis [5]. And strikingly, exogenous miR-30c-5p delivered by recombinant adeno-associated virus (rAAV) markedly ameliorated abnormal triglyceride accumulation and liver steatosis in db/db mice, suggesting a new therapeutic strategy against NAFLD.

## RESULTS

### Decreased miR-30c-5p expression and increased triglyceride accumulation was detected in db/db mice liver

**Figure 1:**
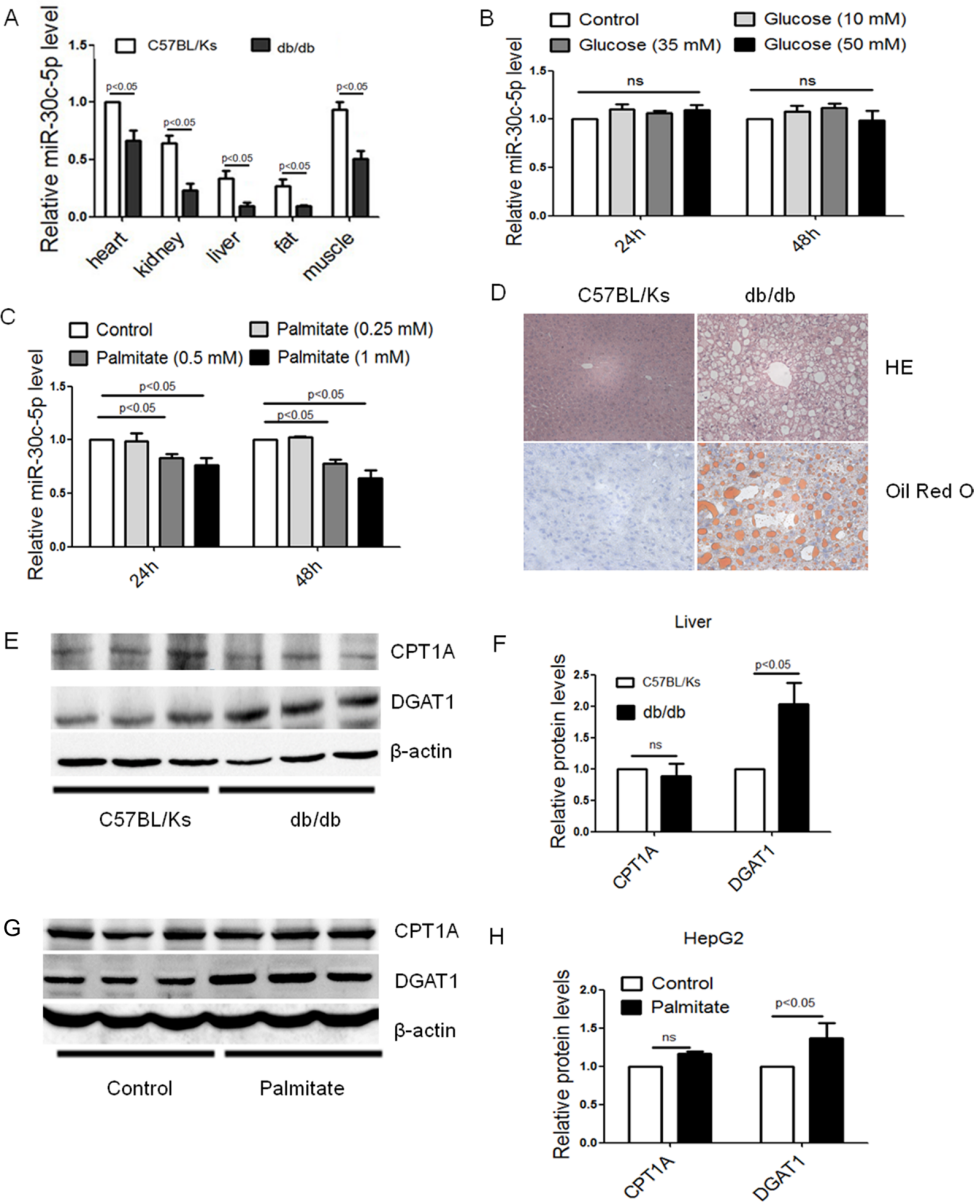
miR-30c-5p expression and triglyceride accumulation in db/db mice (**A**) Relative expression of miR-30c-5p among different organs. (**B**) Relative expression of miR-30c-5p in glucose-treated HepG2 cells. (**C**) Relative expression of miR-30c-5p in palmitate-treated HepG2 cells. (**D**) Histological analysis of hepatocyte by H&E and Oil Red O staining. (**E** and **F**) Protein levels of CPT-1A and DGAT1 in liver of db/db mice and normal controls. (**G** and **H**) Protein levels of CPT-1A and DGAT1 in HepG2 cells.

To explore the role of miR-30c-5p in NAFLD, real-time PCR was used to detect miR-30c-5p expression in db/db mice, a classic animal model for NAFLD. Compared with C57BL/Ks controls, significantly decreased miR-30c-5p was observed in liver of 24-week-old db/db mice (Figure 1A). Notably, miR-30c-5p was also decreased in other tissues of db/db mice. To further determine whether the decreased miR-30c-5p was hyperglycemia- or hyperlipidemia-dependent, cultured HepG2 cells were treated with high glucose and palmitate, respectively. Interestingly, high glucose treatment did not induce change of miR-30c-5p level, while palmitate significantly inhibited miR-30c-5p expression in HepG2 cells (Figure 1B and 1C).

Meanwhile, hematoxylin and eosin (HE) staining and Oil Red O staining showed excessive lipid accumulation and hepatic steatosis in db/db mice (Figure 1D). Interestingly, Western blotting suggested that diglyceride acyltransferase 1 (DGAT1), a crucial and terminal enzyme for triglycerides synthesis, increased significantly in db/db mice liver (Figure 1E and 1F). However, carnitine palmitoyltransferase IA (CPT-1A), the essential and rate-limiting enzyme in the beta-oxidation of long chain fatty acids, showed no differences between two groups (Figure 1E and 1F). Consistent with the *in vivo* study, increased DGAT1 but unaltered CPT-1A were also observed in palmitate treated HepG2 cells (Figure 1G and 1H).

All these data can lead to a hypothesis that decreased miR-30c-5p level might accelerate the abnormal lipid metabolism of db/db mice.

### rAAV-miR-30c-5p decreased plasma triglyceride in db/db mice

**Figure 2:**
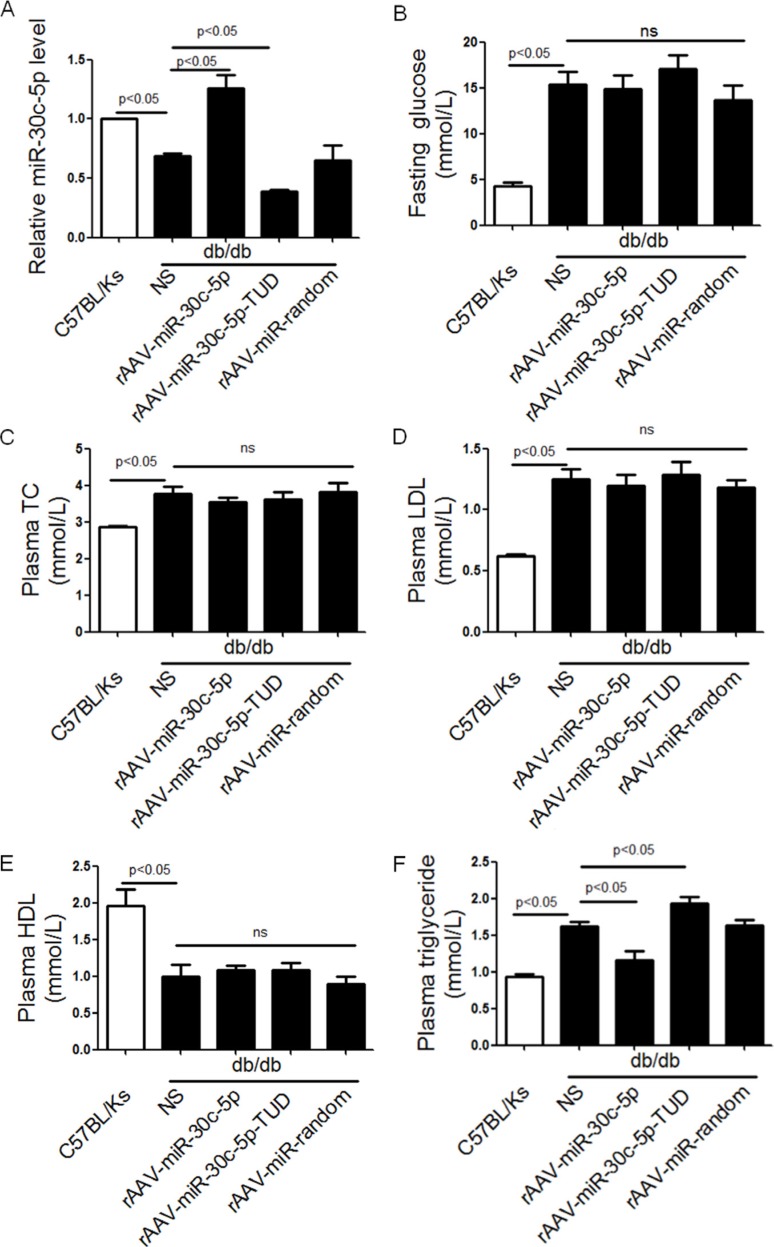
rAAV-miR-30c-5p decreased plasma triglyceride in db/db mice (**A**) Relative expression level of miR-30c-5p in liver of treated mice. (**B**–**F**) Fasting glucose, Plasma TC, LDL, HDL and triglyceride in treated mice.

In order to investigate our hypothesis above, we next employed the db/db mice model to determine the effects of miR-30c-5p on NAFLD by using rAAV delivery system. Twelve-week–old male db/db mice were divided into 4 groups (*n* = 8 in each) and treated with NS (control saline), rAAV-miR-30c-5p, rAAV-miR-30c-5p-TUD, and rAAV-miR-random, respectively, for 12 weeks. Results showed that hepatic miR-30c-5p was significantly decreased in db/db mice compared to C57BL/Ks controls, while treating with rAAV-miR-30c-5p-TUD aggravated this decrease in liver of db/db mice. On the contrary, comparing with db/db controls or C57BL/Ks controls, increased hepatic miR-30c-5p was observed in rAAV-miR-30c-5p treated db/db mice (Figure 2A). The level of blood glucose in db/db mice was much higher than C57BL/ks mice, but was not changed by miR-30c-5p over-expression (Figure 2B).

Further, the effects of miR-30c-5p on plasma lipids were detected. Compared with C57BL/Ks controls, increased plasma total cholesterol (TC) and low-density lipoprotein (LDL), but decreased high-density lipoprotein (HDL) was observed in db/db mice. Interestingly, compared with untreated db/db mice, fasting glucose, TC, HDL and LDL remained unaltered in rAAV-miR-30c-5p or rAAV-miR-30c-5p-TUD treated db/db mice (Figure 2B–2E). However, overexpression of miR-30c-5p significantly attenuated plasma triglyceride accumulation in db/db mice, while rAAV-miR-30c-5p-TUD aggravated it (Figure 2F).

Conclusively, miR-30c-5p decreased only plasma triglyceride level, but not glucose or cholesterol in db/db mice.

### rAAV-miR-30c-5p attenuated hepatic steatosis in db/db mice

**Figure 3:**
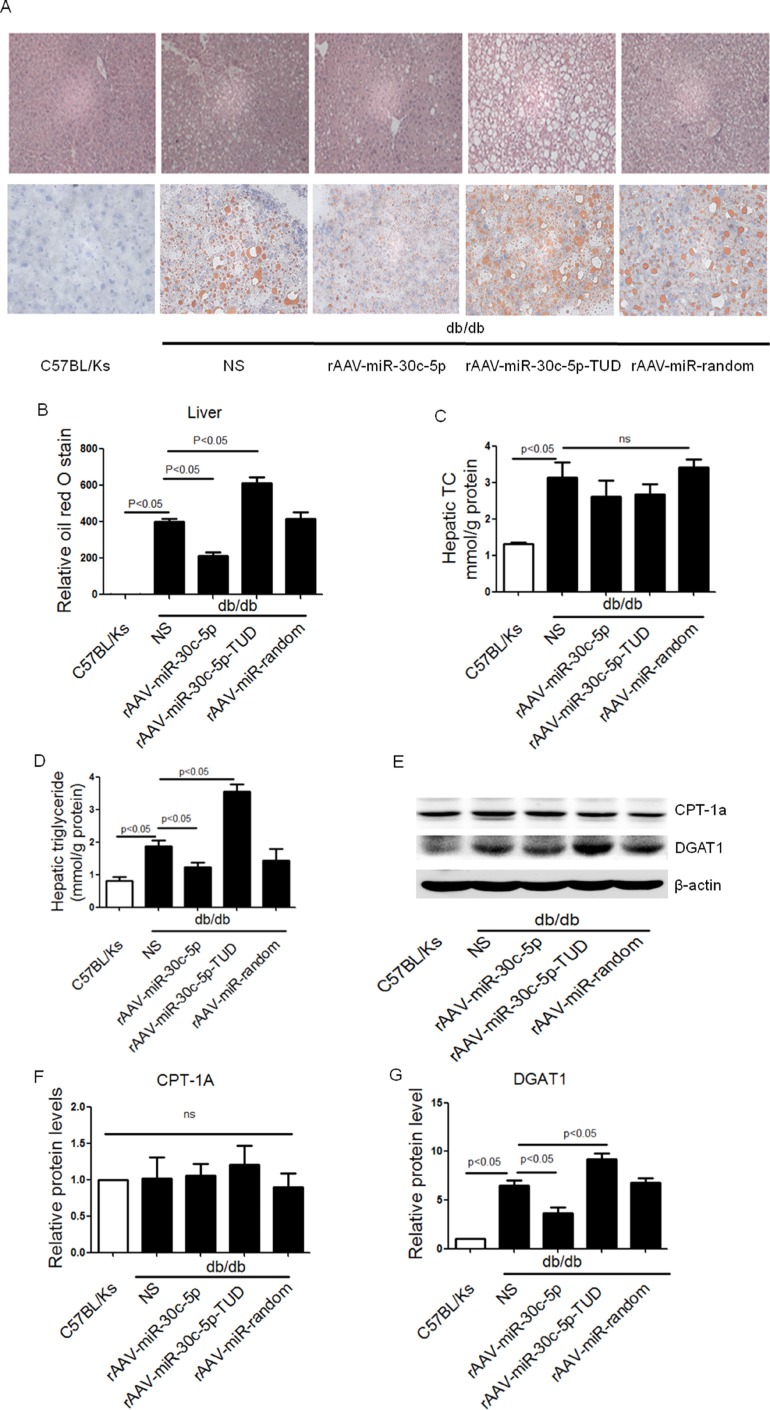

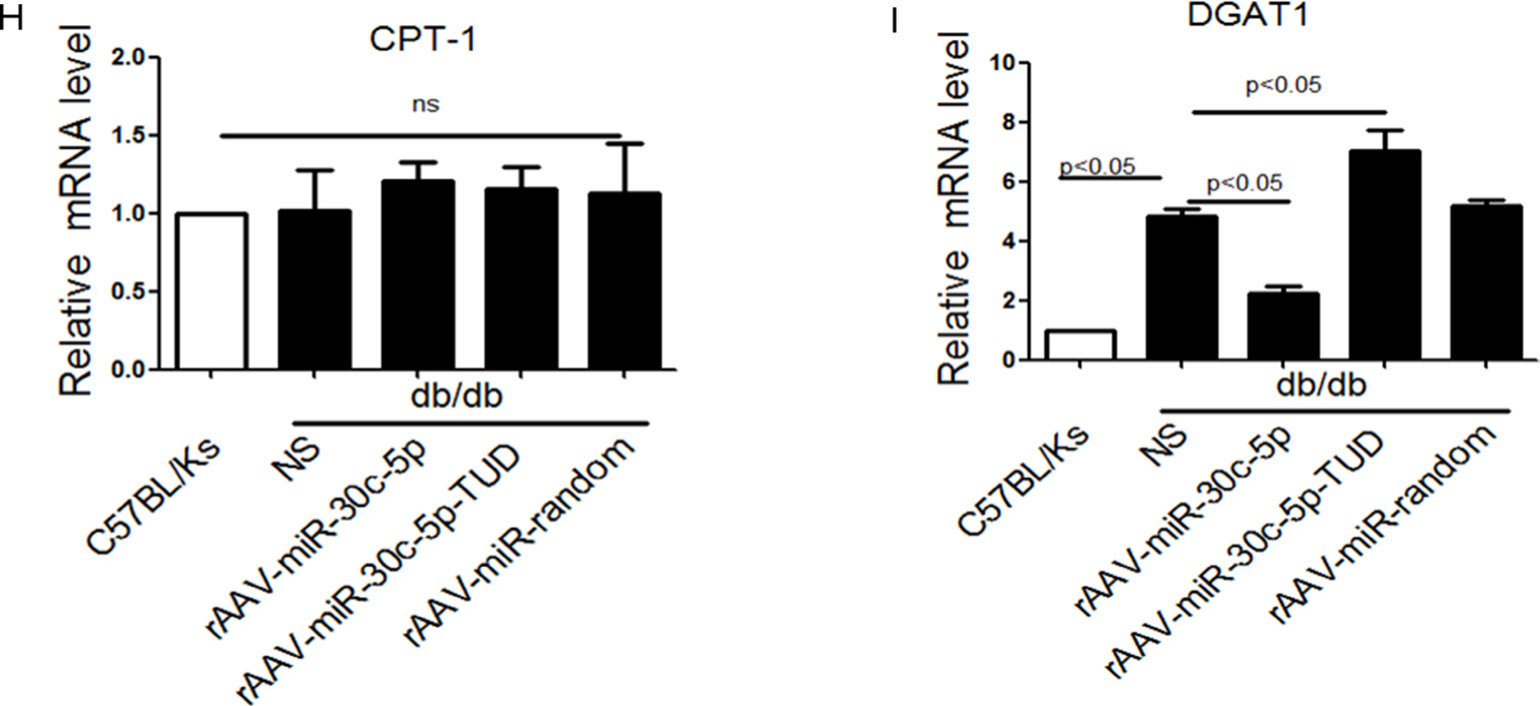
rAAV-miR-30c-5p attenuated hepatic steatosis in db/db mice (**A** and **B**) Histological analysis of hepatocyte by H&E and Oil Red O staining. (**C**) Hepatic TC in treated mice. (**D**) Hepatic triglyceride in treated mice. (**E**–**G**) Protein levels of CPT-1A and DGAT1 in liver of treated mice. (**H** and **I**) Relative mRNA levels of CPT-1A and DGAT1 in liver of treated mice.

Compared with C57BL/Ks controls, excessive lipid accumulation was observed in liver of db/db mice. rAAV-miR-30c-5p treatment significantly attenuated hepatic lipid deposition and hepatic steatosis in db/db mice compared with untreated db/db mice, while rAAV-miR-30c-5p-TUD further aggravated these (Figure 3A and 3B). Consistent with the data observed in plasma, hepatic TC remained unaltered with either rAAV-miR-30c-5p or rAAV-miR-30c-5p-TUD treatment (Figure 3C). However, rAAV-miR-30c-5p treatment reduced hepatic triglyceride accumulation in db/db mice, while rAAV-miR-30c-5p-TUD administration aggravated it (Figure 3D).

Further, we analyzed the protein and mRNA levels of CPT-1A and DGAT1, the crucial enzymes for triglyceride oxidation and synthesis, respectively. Interestingly, we observed a significantly decreased DGAT1 expression in rAAV-miR-30c-5p treated db/db mice, while CPT-1A remained unaltered among all groups (Figure 3E–3I).

These data indicated that miR-30c-5p attenuated hepatic triglyceride accumulation in db/db mice, possibly by reducing triglyceride synthesis.

### miR-30c-5p attenuated palmitate induced triglyceride accumulation *in vitro*

**Figure 4:**
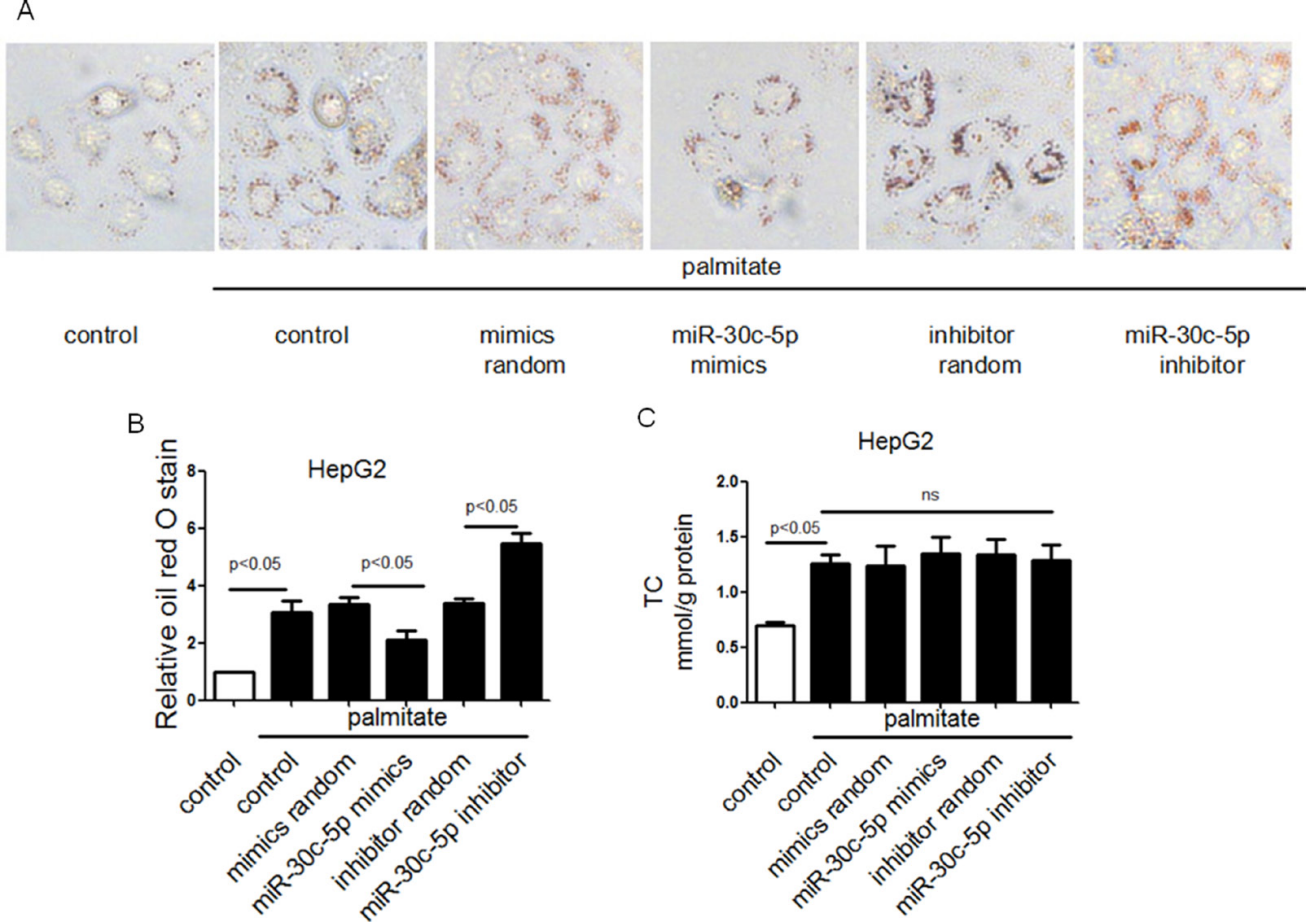

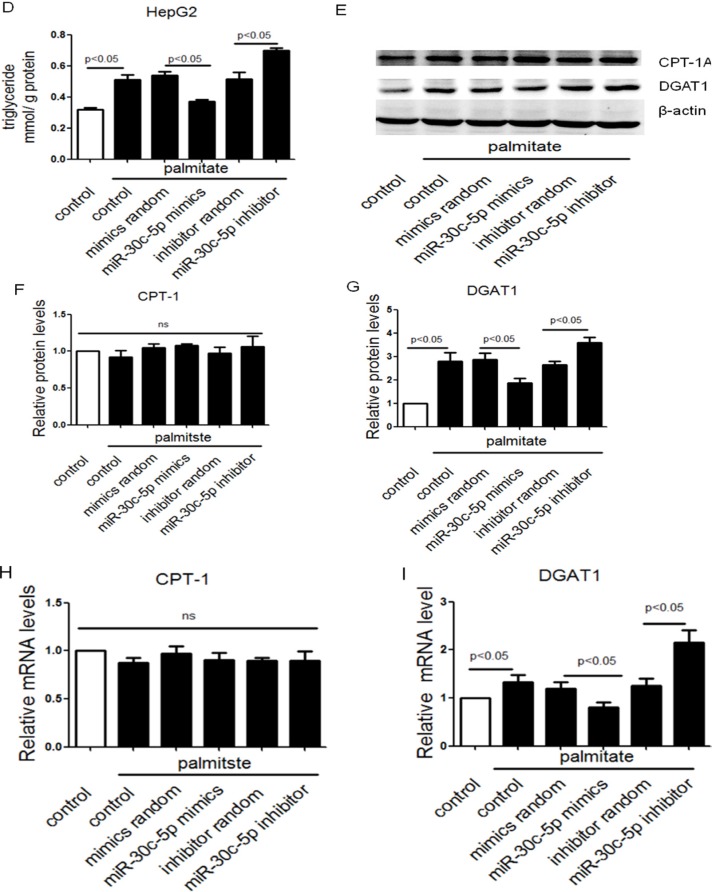
miR-30c-5p attenuated palmitate induced triglyceride accumulation *in vitro* (**A** and **B**) Oil Red O staining analysis of HepG2 cells. (**C**) TC accumulation in HepG2 cells. (**D**) Triglyceride accumulation in HepG2 cells. (**E**–**G**) Protein levels of CPT-1A and DGAT1 in HepG2 cells. (**H** and **I**) Relative mRNA levels of CPT-1A and DGAT1 in HepG2 cells.

To explore the effect of miR-30c-5p on triglyceride accumulation *in vitro*, palmitate-treated HepG2 cells were used as a cell model of lipid-overload hepatic steatosis. As expected, increased lipid deposition was observed in palmitate-treated HepG2 cells. Besides, gain/loss-of-function analysis was conducted by transfection of miRNA mimics or inhibitor. MiR-30c-5p mimics attenuated palmitate-induced lipid accumulation in HepG2 cells, while miR-30c-5p inhibitor aggravated it (Figure 4A and 4B). Increased TC was observed in palmitate-treated HepG2 cells, but remained unaltered with either miR-30c-5p mimics or inhibitor treatment (Figure 4C). More importantly, palmitate-treated HepG2 with miR-30c-5p transfection exhibited reduced triglyceride accumulation while miR-30c-5p inhibitor treatment aggravated it (Figure 4D).

We detected the protein and mRNA levels of CPT-1A and DGAT1. We also found that, *in vitro*, miR-30c-5p mimics treatment decreased DGAT1 expression, while miR-30c-5p inhibitor increased DGAT1 level. However, bioinformatic prediction and Ago2-RIP showed that DGAT1 was not a direct target of miR-30c-5p (Supplementary Figure 1A and 1B). Besides, the protein and mRNA levels of CPT-1A was unaltered among all groups (Figure 4E–4I).

These findings further indicated that miR-30c-5p may attenuate triglyceride accumulation by reducing lipid biosynthesis, but not directly targeting DGAT1.

### miR-30c-5p suppressed FASN level

**Figure 5:**
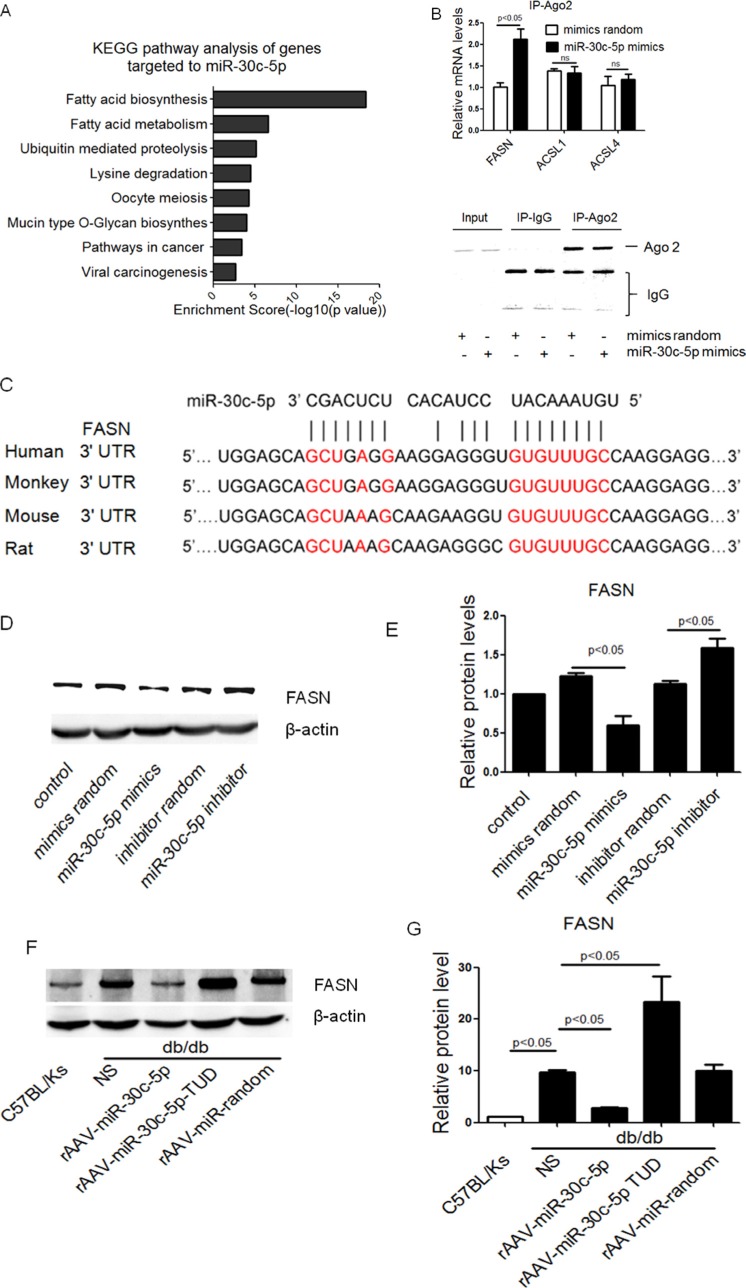
miR-30c-5p suppressed FASN expression (**A**) KEGG pathway analysis of genes targeted by miR-30c-5p. (**B**) Relative mRNA levels detected by Ago2-RIP and western blotting analysis of Ago2-RIP products using anti-Ago2 antibody. (**C**) Schematic representation of the predicted target sites of miR-30c-5p in 3′ UTR of FSAN. (**D** and **E**) Protein level of FASN in HepG2 cells. (**F** and **G**) Protein level of FASN in treated mice.

To understand possible mechanisms through which miR-30c-5p reduces triglyceride metabolism, target genes of miR-30c-5p were predicted using DIANA-mirPath v3.0 Tools. As a result, 1564 genes were potential targets of miR-30c-5p. Furthermore, KEGG pathway analysis was applied to the target pool (1564 genes) to better understand the role of miR-30c-5p. Interestingly, top 8 pathways-suppressed by miR-30c-5p were fatty acid biosynthesis, fatty acid metabolism, ubiquitin mediated proteolysis, lysine degradation, oocyte meiosis, mucin type O-Glycan biosynthesis, pathways in cancer and viral carcinogenesis (Figure 5A). Specifically, three genes, fatty acid synthase (FASN), long chain fatty acid CoA ligase 1 m (ACSL1), and long chain fatty acid CoA ligase (ACSL4) in the fatty acid biosynthesis pathway (the top 1 predicted pathway) were suggested as targets of miR-30c-5p.

To determine the putative target of miR-30c-5p, we performed RNA co-immunoprecipitation with anti-Ago2 antibodies and results showed that miR-30c-5p transfection increased mRNA level of FASN rather than ACSL1 or ACSL4 (Figure 5B). Moreover, miR-30c-5p also showed a potential to target FASN in a highly conserved region among human, monkey, rat, and mouse (Figure 5C). In HepG2 cells, miR-30c-5p transfection decreased FASN level, while knocking down of endogenous miR-30c-5p showed opposite effect (Figure 5D and 5E). Western blotting revealed that hepatic FASN protein level was increased in db/db mice compared with C57BL/Ks control (Figure 5F and 5G), but rAAV9-miR-30c-5p-treatment reduced its expression, while rAAV9-miR-30c-5p TUD treatment showed the opposite effect (Figure 5F and 5G).

These data indicated that miR-30c-5p inhibited fatty acid biosynthesis possibly by suppressing FASN.

### Down-regulation of FASN by siRNA attenuated triglyceride accumulation

**Figure 6:**
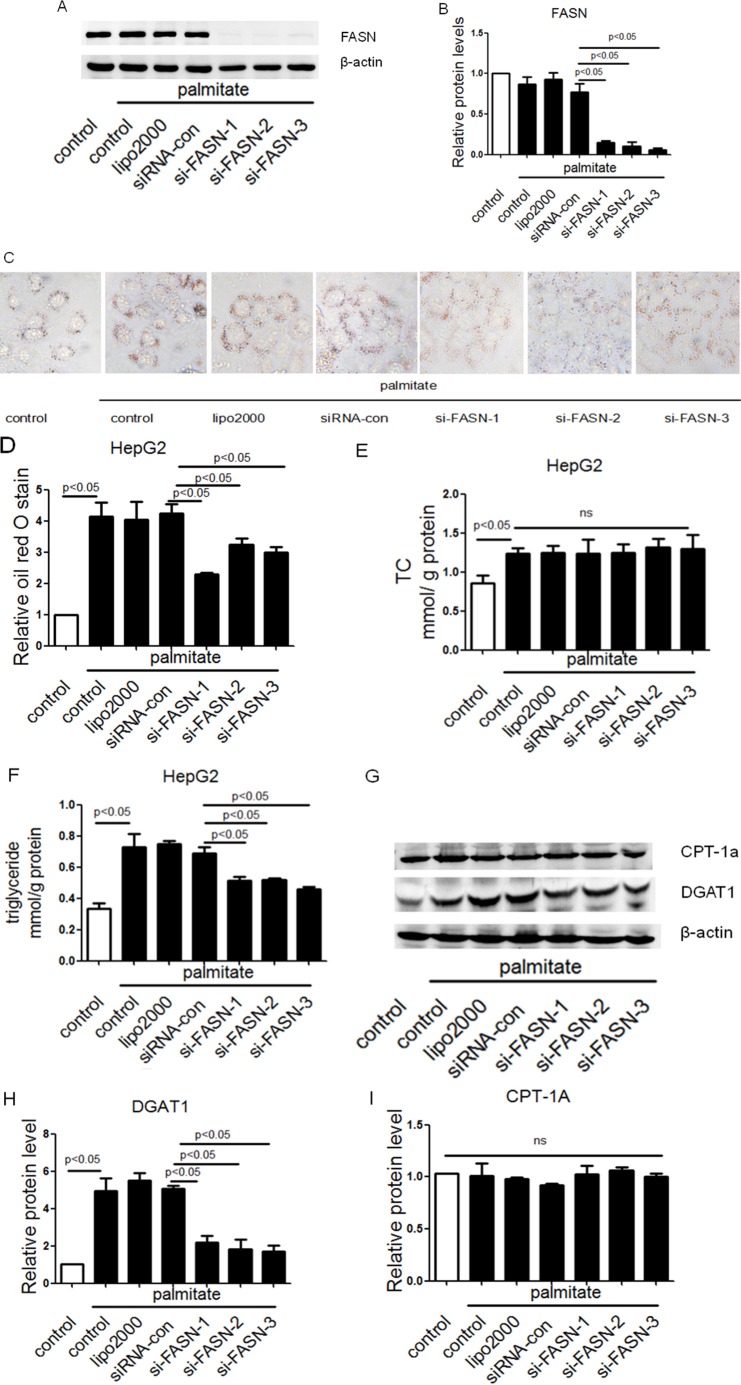

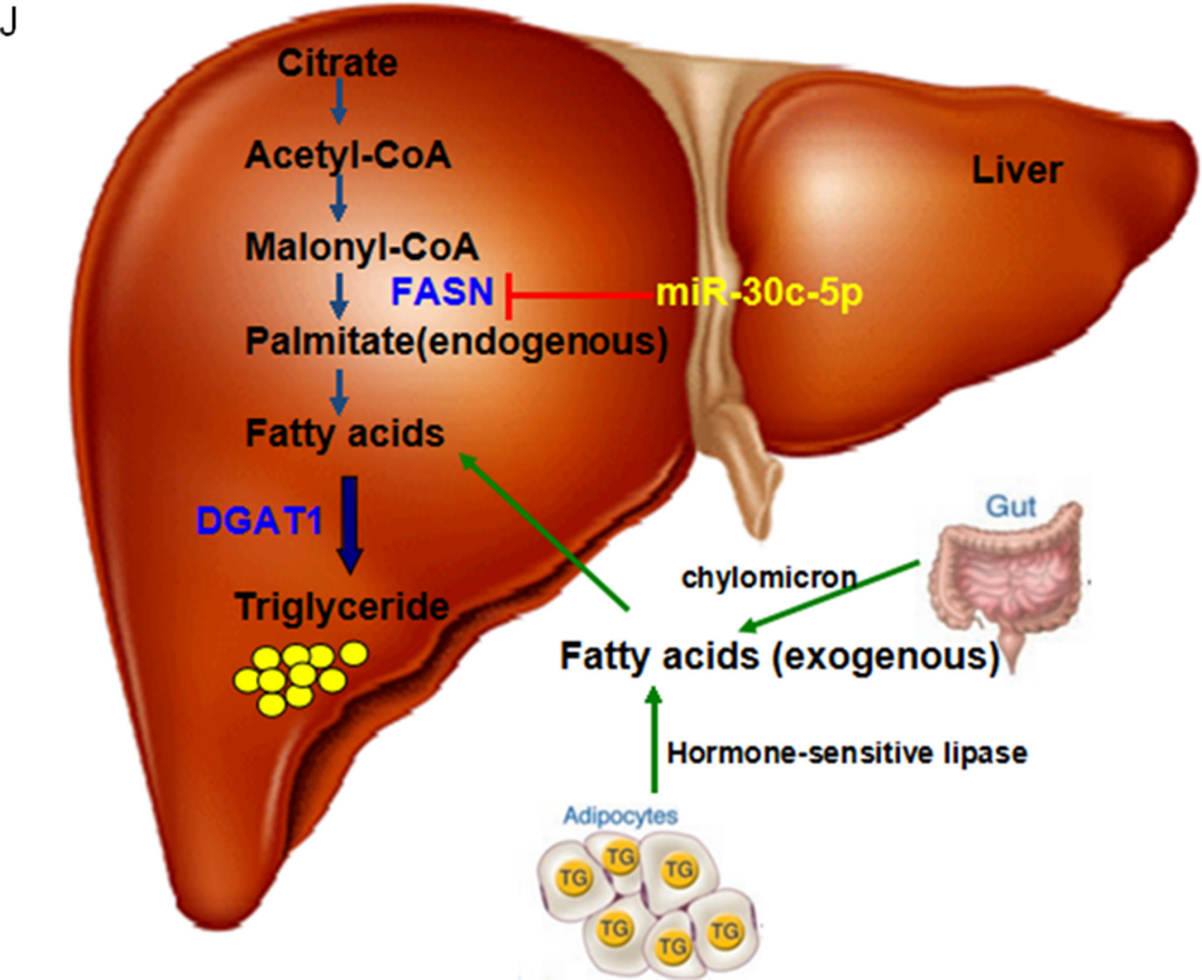
Down-regulation of FASN by siRNA attenuated triglyceride accumulation (**A** and **B**) Protein level of FASN in HepG2 cells. (**C** and **D**) Oil Red O staining analysis of HepG2 cells. (**E**) TC accumulation in HepG2 cells. (**F**) Triglyceride accumulation in HepG2 cells. (**G**–**I**) Protein levels of CPT-1A and DGAT1 in HepG2 cells. (**J**) A model to illustrate the role of FASN and miR-30c-5p.

To verify enhancing function of FASN in palmitate-induced triglyceride accumulation in cultured HepG2 cells, siRNAs against FASN (si-FASN-1, si-FASN-2, si-FASN-3) were transfected into HepG2 cells and more than 80% FASN was knockdowned at protein level (Figure 6A and 6B). The knockdown of FASN reversed lipid deposition and triglyceride accumulation, but TC level remained unchanged in palmitate treated HepG2 cells (Figure 6C–6F). Furthermore, Western blotting confirmed a decrease in DGAT1 but not CPT1A in si-FASN treated HepG2 cells (Figure 6G–6I). These data indicated that knockdown of FASN attenuated triglyceride accumulation, which is in consistence with the effects of miR-30c-5p overexpression.

Furthermore, a model was employed to illustrate the roles of FASN and miR-30c-5pin hepatic steatosis (Figure 6J).

## DISCUSSION

In the present study, we observed down-regulation of the miR-30c-5p in db/db mice, which contributed to hepatic steatosis. We found that miR-30c-5p was able to suppress FASN, the crucial enzyme in fatty acid biosynthesis and delivery of exogenous miR-30c-5p by rAAV was sufficient to attenuate triglyceride accumulation and liver steatosis in the db/db mice, suggesting a new therapeutic strategy against NAFLD.

Using DIANA-mirPath v3.0 tool, we identified 1564 genes targeted by miR-30c-5p. KEGG analysis of these target genes further indicated fatty acid biosynthesis as the most possible pathway inhibited by miR-30c-5p. Consistent with this pathway analysis, our *in vivo* and *in vitro* study confirmed down-regulation of miR-30c-5p on fatty acid biosynthesis. Though our KEGG analysis also revealed other pathways (such as cancer) likely to be suppressed by miR-30c-5p, the high enrichment score of fatty acid biosynthesis strongly suggested miR-30c-5p as a metabolic-related miRNA.

As observed in the present work, augmented fatty acid biosynthesis leads to hepatic lipid deposition and steatosis in db/db mice, a classic animal model for NALFD [16]. Delivery of exogenous miR-30c-5p efficiently attenuated triglyceride accumulation and liver steatosis in the db/db mice. We then asked whether miR-30c-5p was crucial in regulating fatty acid biosynthesis, as hundreds of other miRNAs might also suppress this particular pathway. Using the “Reverse Search module” of DIANA-mirPath v3.0 tool, 57 miRNAs were identified to suppress at least 2 genes in the fatty acid biosynthesis pathway (Supplementary Table 1). Though miR-30c-5p was just 1 of the 57 fatty acid biosynthesis–related miRNAs, its high abundance (one of the top 10 highest expressed hepatic miRNAs [17]) may render it as a more potential regulator compared with other NALFD-related miRNAs.

FASN, ACSL1 and ACSL4 in the fatty acid biosynthesis were specifically suggested to be the putative targets of miR-30c-5p based on bioinformatic analysis, but Ago2-RIP identified only FASN (but not ACSL1 or ACSL4) as the putative targets of miR-30c-5p. MiR-30c-5p decreased FASN expression and triglyceride accumulation, while down-regulation of FASN by siRNA reduced triglyceride accumulation and lipid deposition, which suggested that miR-30c-5p might attenuate fatty acid biosynthesis by targeting FASN.

CPT-1A is a essential and rate-limiting enzyme in the beta-oxidation of long chain fatty acids, but our results showed that the protein and mRNA levels of CPT-1A in the liver of db/db mice and HepG2 cells were unaltered when miR-30c-5p over-expressed. Previous studies in humans and rodents have showed that the mechanisms leading to the excessive triglyceride accumulations in liver were mainly associated with enhanced delivery of plasma none-sterified fatty acid from peripheral tissues to liver and increased hepatic de novo lipid synthesis, while lipid degradation was only moderately inhibited through β-oxidation and secretion of VLDL into plasma [18]. The present study also indicated that miR-30c-5p reduced novo lipid synthesis in hepatic steatosis.

DGAT1, which catalyzes the last step in hepatic triglyceride synthesis, is enriched in human liver and increased in patients with NAFLD [19, 20]. It aggravates the development of hepatic steatosis via enhancing esterification of exogenous fatty acids but not endogenous fatty acids [21]. This study demonstrated that miR-30c-5p down-regulated DGAT1 expression and therefore reduced exogenous fatty acids esterification to prevent hepatic steatosis. However, another research showed that deficiency of DGAT1 did not protect db/db mice against hepatic steatosis, which indicated that excessive triglyceride accumulations in liver of db/db mice may result from not only enhanced exogenous, but also endogenous fatty acids synthesis [22]. Therefore, inhibiting endogenous fatty acids synthesis may be a potential therapy for hepatic steatosis, which is supported by our study.

Endogenously-synthesized fatty acids are catalyzed by the 250-270 kD multifunctional, homodimeric FASN. FASN synthesizes long-chain fatty acids by using acetyl-CoA as a primer, malonyl-CoA as a two-carbon donor, and NADPH as a reducing equivalent. The predominant product of FASN is a 16-carbon fatty acid, palmitate [23–25]. Interestingly, previous studies have demonstrated that treatment of FASN inhibitor in obese mice caused dramatic improvement of hepatic steatosis [26, 27], which was consistent with our results.

Our data suggested that knockdown of FASN attenuated triglyceride accumulation in palmitate-treated HepG2 cells, which was in consistence with the effects of miR-30c-5p overexpression. The explanation may be as follows: 1) Exogenous and endogenous palmitate together contributed to cellular triglyceride accumulation; 2) Si-FASN decreased endogenous palmitate synthesis, which may further lead to attenuated triglyceride synthesis; 3) In addition to catalyzing palmitate synthesis, FASN may also inhibit other fatty acid biosynthesis-related enzymes such as DGAT1 to reduce exogenous fatty acids esterification, which was observed in our study and previous works [27]. Taken together, miR-30c-5p may attenuate triglyceride accumulation via FASN or FASN-regulated DGAT1 directly.

rAAV system, with low toxicity and antigenicity, is a promising vector for gene therapy [12, 28]. Previous researches showed a preference for liver tissue rAAV9 being clearly superior in terms of transduction efficiency and transgene expression compared to rAAV2 and rAAV8 [29]. Thus rAAV9 was used for the delivery of miR-30c-5p in the present study. However, rAAV delivered miR-30c-5p also target other organs, such as heart and kidney, and thus the potential off-target effects of miR-30c-5p in the current study could not be completely ruled out.

In summary, our findings reveal an inhibitory role of miR-30c-5p in NALFD, and its systematic delivery to animals is sufficient to reduce triglyceride accumulation and alleviate hepatic steatosis in db/db mice by reducing both endogenous fatty acids synthesis and exogenous fatty acids esterification. These observations provide a theoretical basis for developing miRNA-based therapeutics against metabolism disorder and associated diseases.

## MATERIALS AND METHODS

### Cell culture and transfection

HepG2 cells and HEK293 cells were maintained in DMEM with10% FBS (Life Technologies, Carlsbad, CA). MiRNA mimics, inhibitors, siRNAs, and random small RNA controls were transfected by Lipofectamine 2000 (Life Technologies, Carlsbad, CA). All of the small RNAs used in the present study were purchased from Riobio Co., Ltd (Guangzhou, China).

### Protein extraction and western blotting

Protein concentrations were determined by the BCA method. For Western blotting, total cell lysate was resolved by SDS-PAGE, transferred to PVDF membrane, and blocked with 5% non-fat dry milk in TBS-T. The membrane was incubated with primary antibody overnight at 4°C, followed by peroxidase-conjugated secondary antibody for 2 hours, and finally developed with the ECL system (Beyotime Institute of Biotechnology, Nanjing, China). Antibodies used in the present study were from ABclonal technology (Boston, MA): CPT1A (Catalog No: A5307), DGAT1 (Catalog No: A6857), FASN (Catalog No: A6273), and β-actin (Catalog No: AC004). Western blotting results were quantified by densitometry and processed with the ImageJ software (National Institutes of Health software).

### RNA extraction and quantitative RT-PCR

Total RNA was isolated using TRIzol (Life Technologies, Carlsbad, CA) and reverse transcribed with First Strand Synthesis Kit (Life Technologies, Carlsbad, CA). Real-time PCR were performed with the SYBR Green (Life Technologies, Carlsbad, CA) on a 7900HT FAST Real-Time PCR System (Life Technologies, Carlsbad, CA) at 95°C for 10 min, 40 cycles at 95°C for 15 s in each cycle, and 60°C for 1 min in the final cycle. All reactions were performed in triplicate. Primers used in the present study were listed in Supplementary Table 2.

### RNA immunoprecipitation

Lysed cell extracts were immunoprecipitated with anti-Ago2 antibody (Abnova Corporation, Taiwan, China) or IgG (Santa CruzBiotechnology, Santa Cruz, CA) using protein G Sepharose beads (Santa Cruz Biotechnology, Santa Cruz, CA), as described [30]. After elution from the beads, bound RNA were extracted with TRIzol and quantified by real time RT-PCR.

### Construction of rAAV

rAAV (type 9) containing miR-30c-5p, miR-30c-5p-TUD, or miR-random were prepared by triple plasmid co-transfection in HEK293T cells, respectively, as previously described [28, 30].

### Animals

All experiments were performed with the approval of the Animal Research Committee of Tongji Medical College, and in accordance with ARRIVE and NIH guidelines for animal welfare. For *in vivo* experiments, male db/db mice on C57BL/Ks background and control C57BL/Ks mice (Model Animal Research Center of Nanjing University, Nanjing, China) were used. All the animals were maintained with 12-h light/12-h dark photoperiods with free access to water and food. We randomly divided db/db mice into four groups (control, rAAV-miR-random, rAAV-miR-30c and rAAV-anti-miR-30c, *n* ≥ 8 each group). They were injected with corresponding rAAVs via tail vein at the age of 12 weeks. All surgery was performed under sodium pentobarbital anesthesia to minimize suffering. Through intraperitoneal injections of a ketamine (80 mg/kg) and xylazine (5 mg/kg) mixture, anaesthetization of mice was performed. To assess the adequacy of anesthesia during hemodynamic examinations, parameters such as responsiveness, blood pressure, respiratory and heart rates were monitored. Then they were sacrificed by CO_2_ inhalation after the surgical procedures. The rAAV-treated db/db and control C57BL/Ks mice were sacrificed at 24 weeks and tissue samples were snap-frozen in liquid nitrogen or collected for paraffin embedding.

### Histological analysis

Formalin-fixed livers were embedded in paraffin and sectioned into 4 mm slices. The morphology was detected by HE staining. Oil Red O staining was applied to frozen, 7 μm sections. Lipid deposition were visualized by microscope, and measured by Image-Pro Plus Version 6.0 (Media Cybernetics, Bethesda, MD).

### Biochemical parameters

TC, triglyceride, LDL, and HDL in plasma were measured on an AEROSET Clinical Chemistry System (Abbott Laboratories). TC and triglyceride in liver or HepG2 cells were detected by GRO-PAP method (Nanjing Jiancheng Bioengineering Institute, Nanjing, China).

### MiRNA targets and pathway analysis

DIANA-mirPath v3.0, a miRNA pathway analysis web-server, was utilized for target prediction of miR-30c-5p. MirPath can utilize predicted miRNA targets (in 3′ UTR regions) provided by the experimentally validated miRNA interactions derived from DIANA-TarBase v7.0. These interactions can be subsequently combined with sophisticated merging and meta-analysis algorithms [31]. Furthermore, the KEGG analysis was performed for pathway analysis of all miR-30c-5p targets.

### Statistics

All data are presented as mean ± SEM. The Student's *t* test and ANOVA were performed, to determine statistically significant differences among treatment groups, as appropriate. In all cases, a value of *p* < 0.05 was considered to be statistically significant.

## SUPPLEMENTARY MATERIALS FIGURE AND TABLES


