# Supplementary material for: MiR-30c-5p ameliorates hepatic steatosis in leptin receptor-deficient (db/db) mice via down-regulating FASN
Source: Oncotarget. 2017 Jan 9;8(8):13450–63. doi: 10.18632/oncotarget.14561 (PMC5355111; doi:10.18632/oncotarget.14561)
Supplement: Supplementary file 1 [file oncotarget-08-13450-s001.pdf]

MiR-30c-5p ameliorates hepatic steatosis in leptin receptor-deficient (db/db) mice via down-regulating FASN

Supplementary Materials

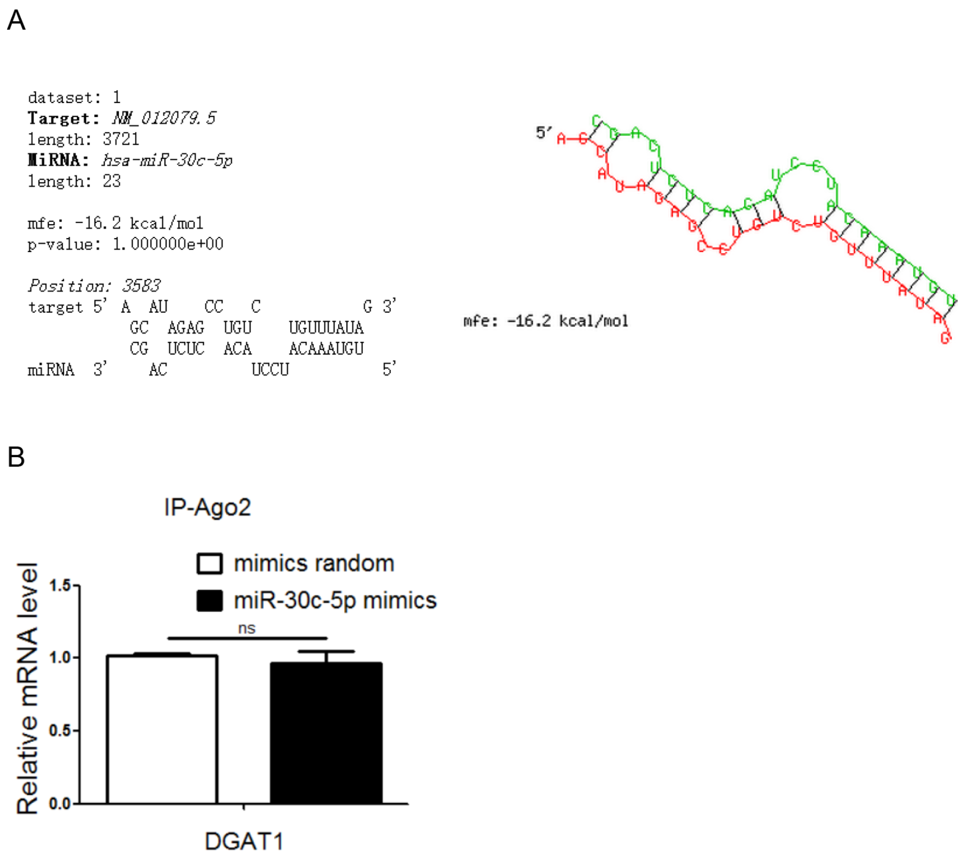

Supplementary Figure 1: DGAT1 was not a direct target of miR-30c-5p. (**A**) The Bielefeld Bioinformatics Server was used to detect the possibility of binding between miR-30c-5p and DGAT1. (**B**) Relative DGAT1 mRNA level detected by Ago2-RIP.

Supplementary Table 1: Fatty acid biosynthesis targeted miRNAs

miRNA	*p*-value	Targeted genes
hsa-miR-34a-5p	7.15E-21	FASN,ACSL1/4/6,ACACA
hsa-miR-15b-5p	2.15E-16	FASN,ACSL3/4,ACACA
hsa-miR-26a-5p	2.15E-16	FASN,ACSL3/4,ACSL1
hsa-miR-15a-5p	2.15E-16	FASN,ACSL3/4,ACACA
hsa-miR-16-5p	2.15E-16	FASN,ACSL3/4,ACACA
hsa-miR-7-5p	2.15E-16	FASN,OXSM,ACSL4,ACACA
hsa-miR-107	2.15E-16	FASN,ACSL3/4,ACSL1
hsa-miR-103a-3p	2.15E-16	FASN,ACSL3/4,ACSL1
hsa-miR-26b-5p	4.2E-12	ACSL3/4,ACSL1
hsa-miR-182-5p	4.2E-12	FASN,ACSL4,ACACA
hsa-miR-522-5p	4.2E-12	FASN,ACSL3,ACACA
hsa-miR-24-3p	4.2E-12	FASN,MCAT,ACACA
hsa-miR-93-5p	4.2E-12	FASN,ACSL3/4
hsa-miR-340-5p	4.2E-12	ACSL3/4,ACACA
hsa-miR-335-5p	4.2E-12	FASN,ACSL3/4
hsa-miR-17-5p	4.2E-12	FASN,ACSL3/4
hsa-miR-27a-3p	4.2E-12	FASN,ACSL3,ACACA
hsa-miR-424-5p	4.2E-12	FASN,ACSL3/4
hsa-miR-130b-3p	5.1E-8	FASN,ACSL4
hsa-miR-877-3p	5.1E-8	FASN,ACACA
hsa-miR-342-3p	5.1E-8	MCAT,ACACA
hsa-miR-1303	5.1E-8	FASN,ACSL4
hsa-miR-101-3p	5.1E-8	FASN,ACSL4
hsa-miR-374a-5p	5.1E-8	ACSL3/4
hsa-miR-30a-5p	5.1E-8	FASN, ACSL4
hsa-miR-148a-3p	5.1E-8	FASN,ACACA
hsa-miR-29a-3p	5.1E-8	FASN,ACACA
hsa-miR-148b-3p	5.1E-8	FASN,ACACB
hsa-miR-221-3p	5.1E-8	ACSL3,ACACA
hsa-miR-19b-3p	5.1E-8	ACSL4,ACACA
hsa-miR-423-3p	5.1E-8	FASN,ACACA
hsa-miR-449a	5.1E-8	ACSL4,ACACA
hsa-miR-30c-5p	5.1E-8	FASN, ACSL4
hsa-miR-19a-3p	5.1E-8	ACSL4,ACACA
hsa-miR-374b-5p	5.1E-8	ACSL3/4
hsa-miR-454-3p	5.1E-8	ACSL3/4
hsa-miR-122-5p	5.1E-8	ACACA,ACACB
hsa-miR-301a-3p	5.1E-8	FASN, ACSL4
hsa-miR-30e-5p	5.1E-8	FASN, ACSL4
hsa-let-7a-5p	5.1E-8	MCAT,ACSL1
hsa-let-7b-5p	5.1E-8	MCAT,ACSL1
hsa-miR-378a-3p	5.1E-8	ACSL4,ACACA
hsa-miR-197-3p	5.1E-8	MCAT,ACACA
hsa-miR-149-5p	5.1E-8	FASN, ACSL4
hsa-miR-548d-3p	5.1E-8	FASN, ACSL4
hsa-miR-100-5p	5.1E-8	FASN, ACSL3
hsa-miR-191-5p	5.1E-8	ACSL1/3
hsa-miR-195-5p	5.1E-8	ACSL4,ACACA
hsa-miR-331-3p	5.1E-8	FASN, ACSL3
hsa-miR-21-5p	5.1E-8	ACSL4/6
hsa-miR-186-5p	5.1E-8	FASN, ACSL4
hsa-miR-20b-5p	5.1E-8	ACSL3/4
hsa-miR-16-1-3p	5.1E-8	ACSL3/4
hsa-miR-106a-5p	5.1E-8	ACSL3/4
hsa-miR-485-3p	5.1E-8	ACSL3,ACACA
hsa-miR-130a-3p	5.1E-8	FASN,ACACA
hsa-miR-375	5.1E-8	ACSL3/4

Supplementary Table 2: List of primers

Gene name	F (5′-3′)	R (5′-3′)
**h-FASN**	ACTACTTTGTGGTCTTCTCCTCTG	TCGTTGGTGCTCATCGTCTC
**h-ACSL 4**	GCTTTTATTCCTGCCCTGCTG	GTGTTCTAGCTTTTGGGAGT
**h-ACSL1**	GGGTTCTGTGAATGCCTGTG	GGGTTCTGTGAATGCCTGTG
**h-DGAT1**	GGGTCCGAGGGTGTCAAT	GGAGTGTGGGGAATGGGG
**h-CPT-1A**	GCCTTTCAGTTCACGGTCAC	CACCACCACGATAAGCCAA
**mus-FASN**	TCCACCTTTAAGTTGCCCTG	TCTGCTCTCGTCATGTCACC
**mus-DGAT1**	GTGGGTTCCGTGTTTGC	TCGGTAGGTCAGGTTGTCT
**mus-CPT1A**	CAAAAAGTAACTGTCGGAGC	CAAGTCAAACGGTGGAGC
